# Variability in clinician intentions to implement specific cognitive-behavioral therapy components

**DOI:** 10.1186/s12888-019-2394-y

**Published:** 2019-12-18

**Authors:** Courtney Benjamin Wolk, Emily M. Becker-Haimes, Jessica Fishman, Nicholas W. Affrunti, David S. Mandell, Torrey A. Creed

**Affiliations:** 1 Department of Psychiatry, Perelman School of Medicine, University of Pennsylvania, 3535 Market Street, Philadelphia, PA 19104 USA; 2 Leonard Davis Institute of Health Economics, University of Pennsylvania, Philadelphia, USA; 3 Hall-Mercer Community Mental Health Center, Philadelphia, PA USA; 4 Annenberg School for Communication, University of Pennsylvania, Philadelphia, USA; 5 National Association of School Psychologists, Bethesda, MD USA

**Keywords:** Cognitive-behavioral therapy, Intentions, Motivation, Implementation, Dissemination

## Abstract

**Background:**

CBT comprises many discrete components that vary in complexity, but implementation and training efforts often approach CBT as a single entity. We examined variability in clinician intentions to use different structural and interventional components of CBT for three different clinical groups: clients receiving CBT, clients with depression, and clients with anxiety.

**Methods:**

Clinicians (*n* = 107) trained in CBT completed a one-time electronic survey. Clinicians’ intentions were measured using established item stems from social psychology adapted to examine intentions to use six specific CBT components: exposure therapy, cognitive restructuring, behavioral activation, planning homework, reviewing homework, and agenda-setting.

**Results:**

Intentions were weakest, on average, for exposure. They were strongest, on average, for reviewing homework. A series of ANOVAs with Tukey’s post-hoc tests revealed that participants intended to use exposure with clients receiving CBT (*p* = .015) and clients with anxiety (*p* < .001) significantly more than for clients with depression. Participants intended to use behavioral activation with clients with depression (*p* = .01) significantly more than for clients with anxiety. No other intentions to use CBT components differed among these three clinical populations.

**Conclusions:**

When studying determinants of CBT use and designing interventions to increase use, implementers should consider that different CBT components may require different implementation strategies.

**Trial registration:**

Not applicable.

## Background

Strong evidence supports using cognitive behavioral therapy (CBT) for a range of mental health problems in children and adults [1, 2]. However, in the U.S. it is rarely is used in routine clinical practice in the community [3, 4]. Implementation strategies, the “methods or techniques used to enhance the adoption, implementation, and sustainability of a clinical program or practice,” [5, 6] aimed at increasing clinicians’ use of evidence-based mental health practices have had limited success to date in increasing the use of CBT [7–10].

CBT’s complexity may contribute to its poor and infrequent implementation [11]. CBT is an overarching term encompassing a set of intervention components guided by cognitive-behavioral theory. To date, most CBT dissemination and implementation efforts have trained clinicians to deliver comprehensive CBT protocols. CBT comprises many discrete components that vary in what they require clinicians to do [12, 13]. CBT involves both structural elements (e.g., agenda-setting, homework assignment, Socratic questioning) and discrete intervention components (e.g., cognitive restructuring, relaxation, exposure). The set of components may vary based on the disorder the clinician is treating. Thus, implementing a “single CBT protocol” requires clinicians to learn multiple components concurrently, which they may use with varying fidelity. A small body of research suggests that clinicians vary in whether and how well they use these components [14] and in how much they value particular components [15]. For example, while exposure is considered a key component in CBT for anxiety [16], community clinicians rarely use it, relying instead on other less effective CBT strategies, such as relaxation [17, 18]. Clinicians may find certain intervention components to be easier to implement, more intuitive, or less aversive (e.g., in the case of exposure) than others, contributing to this variability.

Most approaches to evaluating CBT implementation, as well as studies predicting clinicians’ use of CBT, do not distinguish among CBT’s many components [17]. Recent examination of other psychosocial evidence-based practices (EBPs) suggests that intentions to use specific intervention components, as well as actual use, may vary within and across practitioners, and may call for different implementation strategies for different components [19].

Our research and that of others suggests that intentions are an important, proximal determinant of implementation of evidence-based practices [20, 21]. Intentions are defined as a person’s motivation to perform a behavior, or the effort an individual plans to exert to perform the behavior [22–24]. Clinician’s use of EBPs is the outcome of interest in most implementation studies. In many models of clinician behavior, strong intention is a necessary precursor for behavior change to occur [25]. If the clinician has the skills and resources needed to perform the given behavior, then it is highly likely that he or she will act on those intentions [22–24].

We examined variability in the strength of intentions to use different CBT components, which we think has two important implications. First, if there is variability, it suggests that other measures of clinicians’ thoughts (such as their attitudes or self-efficacy) regarding use of CBT should take this variability into account. Many implementation measures ask clinicians to report their views and use of EBPs broadly instead of their views and use of specific EBP components [26]. Second, variability would suggest that implementation strategies may need to target use of specific components, rather than CBT as a whole. Since intentions may be weaker for certain CBT components, it could be more cost-effective and efficacious to design implementation strategies that target those specific components.

Intentions may be influenced by attitudes (i.e., the perceived advantages and disadvantages of implementing a particular CBT component), perceived norms (e.g., the belief that important others think they should or should not implement the CBT component), and self-efficacy (i.e., confidence in one’s ability to so implement) [23, 27]. Each of these determinants of intention represent potential malleable mechanisms [19]. For example, training and consultation strategies may be sufficient for increasing clinician self-efficacy and fidelity to a component they already strongly intend to use. When intentions to use an intervention component are weak, additional strategies such as policy mandates (to strengthen perceived norms) or financial incentives (to improve attitudes) may be needed to strengthen intentions.

To conduct this study, we surveyed community mental health clinicians who were trained in CBT through the University of Pennsylvania’s Beck Community Initiative (Penn BCI), a large-scale CBT implementation effort conducted in partnership with the Philadelphia Department of Behavioral Health and Intellectual disAbility Services (DBHIDS) [28]. We gathered data on the intentions of community clinicians to use each of six key CBT intervention components (exposure therapy, cognitive restructuring, behavioral activation, planning homework, reviewing homework, and agenda setting). For each CBT component, we gathered data about clinicians’ intentions to use them for three different clinical groups: 1) all clients receiving CBT, 2) clients with depression, and 3) clients with anxiety, because the appropriateness of these components may vary by the presenting problem. We hypothesized that clinicians would have the strongest intentions to use exposure for clients with anxiety, and to use behavioral activation for clients with depression. We also hypothesized that the strength of intentions would not differ across clinical groups for cognitive restructuring, planning homework, reviewing homework, and agenda setting as these CBT strategies are recommended across groups. We hypothesized that intentions to use structural components of CBT (planning homework, reviewing homework, and agenda setting) would be stronger than intentions to use intervention strategies (exposure therapy, cognitive restructuring, behavioral activation) because of their perceived complexity [29].

## Method

### Participants

Our sample comprised 107 clinicians trained in CBT through the Penn BCI [28]. Training consisted of 22 h of content about CBT from foundational through more complex skills, including case conceptualization, intervention components, and relapse prevention, followed by 6 months of weekly group consultation with tape review. Training was conducted either in-person (*n* = 37) or by web (*n* = 70). Clinicians were primarily master’s level (*n* = 88, 82.2%). Eight (6.6%) were doctoral level (i.e., MD or PhD) and four (3.7%) who provided substance use services had a bachelor’s or associate’s degree. Criteria for inclusion in the present study were minimal: participants had to be English speaking and have participated in training or consultation through the Penn BCI. See Table 1.

**Table 1:** Demographic characteristics (*N* = 107)

Characteristic	*n* (%)
Age, mean years (SD)	40.9 (13.4)
Gender	
Male	42 (39.3%)
Female	59 (55.0%)
Missing	6 (5.7%)
Race	
Asian	6 (5.6%)
Black or African American	25 (23.4%)
White	56 (52.3%)
Other	6 (5.6%)
Missing	14 (13.1%)
Hispanic or LatinX	8 (7.5%)
Job Title	
Therapist	69 (64.5%)
Psychologist	9 (8.4%)
Social Worker	7 (6.5%)
Administrator	6 (5.6%)
Creative Arts Therapist	3 (2.8%)
Other	6 (5.6%)
Missing	7 (6.6%)
Time Employed in Current Role	
< 5 years	51 (47.7%)
> 5 years	32 (29.9%)
Missing	24 (22.4%)
Care Setting	
Outpatient	38 (35.5%)
Intensive outpatient (IOP)	14 (13.1%)
School-based services	8 (7.5%)
Residential	5 (4.7%)
Inpatient	9 (8.4%)
Other	33 (30.8%)

We recruited clinicians in two ways, depending on whether they were *currently* receiving training or consultation through the Penn BCI or had *previously* received training or consultation through the Penn BCI. We presented clinicians actively receiving training or consultation with a description of the study while administering standard program evaluation measures. We recruited clinicians who previously received training or consultation through the Penn BCI via email.

### Procedure

The IRB reviewed and approved this project. Between 12/11/2018 and 2/20/2019, participants completed a one-time electronic survey questionnaire that took approximately 5–10 min. The required elements of informed consent were described on the first page of the survey. Individuals agreed to participate by proceeding to complete the questionnaire, which was administered via Research Electronic Data Capture (REDcap), a HIPAA compliant web-based survey platform. Those who completed the questionnaire were entered in a lottery to win one of five $100 gift cards. The survey data were linked with background forms that clinicians completed during their baseline program evaluation through the Penn BCI.

### Measures

#### Intentions

We measured the strength of intentions using validated and widely-used item stems from social psychology that were designed to be adapted to any behavior of interest [23]. We adapted the item stem to measure clinician intentions towards using each of six specific CBT intervention components: “I intend to [perform the specified CBT intervention component for a particular group of clients] over the next 2 or 3 months.” Clinicians responded to each intention statement using a 7-point scale (where 1 = strongly disagree and 7 = strongly agree), with higher numbers representing stronger intentions. Clinicians reported the strength of their intentions to use each of the six specific CBT intervention components for three different clinical groups: 1) all of their clients receiving CBT, 2) clients with depression, and 3) clients with anxiety. We selected these clinical populations because the appropriateness of certain components may vary by presenting problem (anxiety or depression). The six intervention components were selected to capture structural components of CBT that would apply to a wide client population (agenda setting, planning and reviewing homework), discrete intervention components that would apply to a wide client population (cognitive restructuring), and intervention components that are evidence-based for some populations but not others (exposure therapy, behavioral activation). For example, exposure therapy is evidence-based for anxiety but not depression, so we would expect stronger intentions to use exposure therapy for anxiety than to use it for depression. We included a general “clients receiving CBT” group for comparison.

#### Clinician background information

Clinicians completed a 22-item “Personal Information” form upon enrolling in the Penn BCI. This form includes questions about the clinician’s age, gender, race, ethnicity, educational background, years of experience, licensure status, primary clinical responsibilities, theoretical orientation, and CBT experience.

### Data analyses

We cleaned the data by matching background forms with survey responses and screened for outliers by examining histograms and scatterplots of relevant variables. No cases were removed. We used descriptive statistics to describe the sample and variability in intention strength across survey items. We calculated correlations among the intention responses and intraclass correlations (ICC) to estimate how the strength of intention to use each CBT component varied within each clinician across clinical populations. The ICC is a measure of the proportion of variance in intention to use CBT components explained by the individual. We tested whether variability in intention strength differed across the CBT components and clinical groups using one-way analysis of variance (ANOVA).

## Results

### Descriptive statistics and correlations

**Fig. 1:**
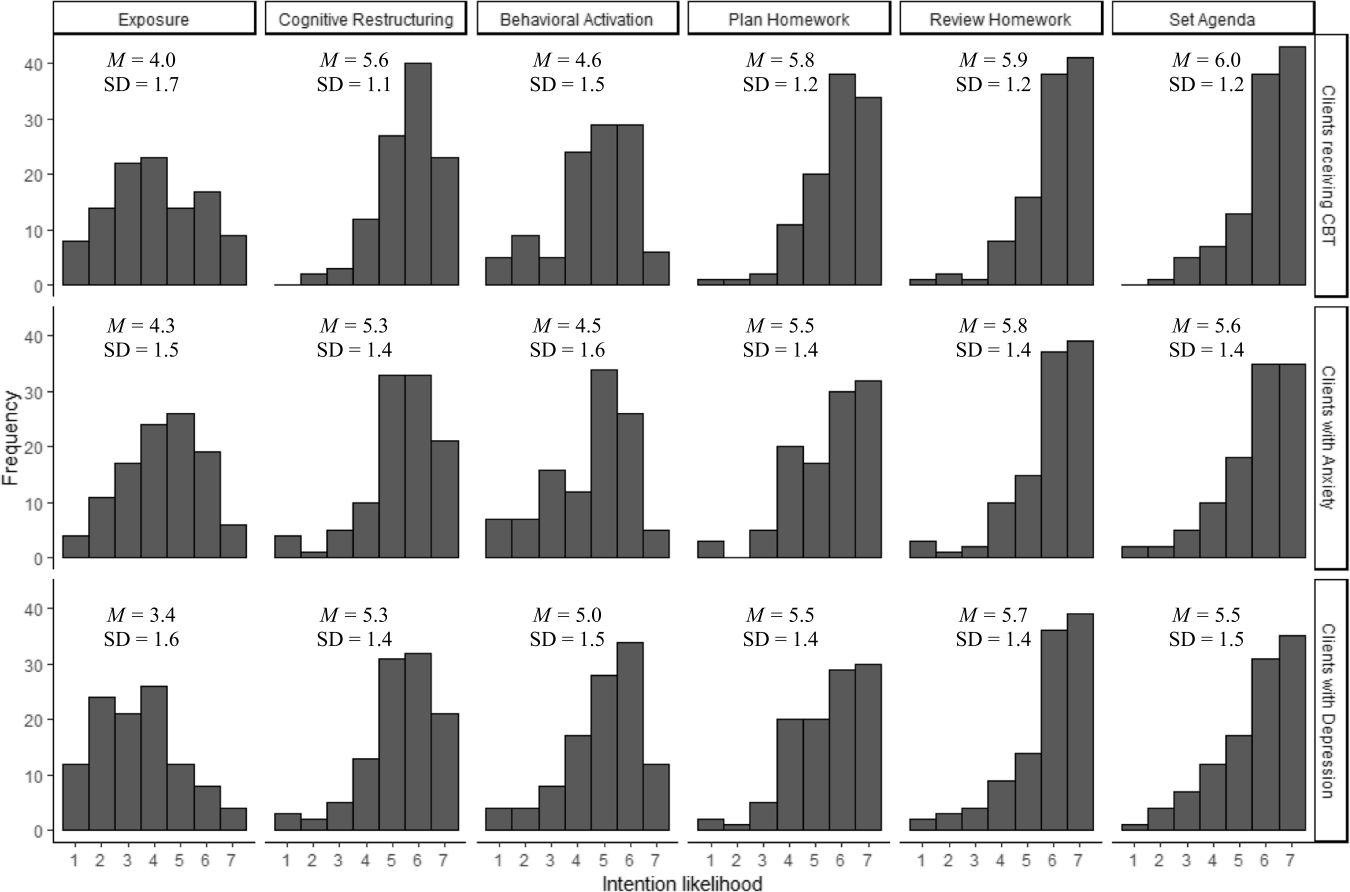
Distribution of CBT component intentions by clinical group

Fig. 1 displays the average strength of intentions to use the CBT components for the three clinical populations. Intentions tended to skew negatively. Intentions were weakest, on average, for exposure (*M* = 3.9), and strongest, on average, for reviewing homework (*M* = 5.8). Across clinical populations, more participants “strongly agreed” that they intended to the use structural components of CBT (i.e., agenda, reviewing homework, and planning homework) than cognitive restructuring; few participants “strongly agreed” that they intended to use behavioral activation and exposure.

Table 2 shows correlations between intentions to use CBT components across the three clinical populations. As expected, there were significant correlations between many pairs of intentions to use CBT components. Intentions towards using structural components of CBT were highly correlated with each other across groups. Intentions towards using CBT interventions (i.e., exposure, cognitive restructuring, and behavioral activation) were moderately correlated with each other and showed mixed associations across and within groups. For example, intentions towards using exposure, on average, showed the lowest correlations with other CBT components, even for clients with anxiety.

**Table 2:** Correlations among intentions to use different CBT components across three clinical groups

		Exposure			Cognitive Restructuring			Behavioral Activation			Plan Homework			Review Homework			Set Agenda		
		CBT	Dep.	Anx.	CBT	Dep.	Anx.	CBT	Dep.	Anx.	CBT	Dep.	Anx.	CBT	Dep.	Anx.	CBT	Dep.	Anx.
Exposure	CBT	–	**.57**	**.55**	**.20**	.12	.17	**.59**	.25	**.41**	.09	.05	.10	.07	−.04	.00	.06	.00	.04
	Depression		–	**.66**	.15	**.32**	**.33**	**.37**	**.30**	**.43**	.03	.13	.18	.07	.10	.12	.09	.16	.19
	Anxiety			–	**.22**	**.39**	**.41**	**.36**	**.45**	**.48**	.13	.16	**.29**	.14	.12	.15	.16	.21	**.27**
Cognitive Restructuring	CBT				–	**.61**	**.63**	**.45**	**.44**	**.31**	**.56**	**.51**	**.46**	**.60**	**.48**	**.51**	**.51**	**.39**	**.39**
	Depression					–	**.93**	.14	**.70**	**.46**	**.43**	**.57**	**.53**	**.47**	**.52**	**.51**	**.39**	**.47**	**.45**
	Anxiety						–	.18	**.67**	**.48**	**.41**	**.54**	**.56**	**.42**	**.46**	**.50**	**.37**	**.42**	**.48**
Behavioral Activation	CBT							–	**.32**	**.55**	**.35**	.23	**.25**	**.27**	.21	.23	.22	.17	.18
	Depression								–	**.59**	**.40**	**.46**	**.44**	**.40**	**.51**	**.49**	**.41**	**.46**	**.46**
	Anxiety									–	.20	**.26**	**.32**	.23	**.26**	**.30**	.22	**.27**	**.31**
Plan Homework	CBT										–	**.82**	**.80**	**.79**	**.73**	**.73**	**.66**	**.52**	**.52**
	Depression											–	**.92**	**.70**	**.82**	**.78**	**.57**	**.67**	**.65**
	Anxiety												–	**.69**	**.77**	**.79**	**.56**	**.64**	**.68**
Review Homework	CBT													–	**.88**	**.89**	**.77**	**.63**	**.64**
	Depression														–	**.96**	**.70**	**.72**	**.71**
	Anxiety															–	**.69**	**.67**	**.72**
Set Agenda	CBT																–	**.81**	**.80**
	Depression																	–	**.95**
	Anxiety																		–

*p* < .05, *p* < .01 entries are in bold

ICCs were high. For intentions to use CBT components with all clients receiving CBT, the ICC = .78. For intentions to use CBT components with clients with depression, the ICC = .83. For intentions to use CBT components with clients with anxiety, the ICC = .83.

### Differences in strength of intentions between groups

Results of the one-way ANOVAs showed a statistically significant difference in the strength of intention to use two of the CBT components across the three clinical groups. There was a statistically significant difference in the strength of intentions to use exposure, F(2, 318) = 8.71, *p* < .001 and behavioral activation F(2, 318) = 3.06, *p* = .048. Tukey’s post-hoc test revealed that participants had stronger intentions to use exposure with clients receiving CBT (*M* = 4.01, *SD* = 1.72, *p* = .015) and clients with anxiety *(M* = 4.29, *SD* = 1.52, *p* < .001) than clients with depression. No difference was observed between the strength of intentions to use exposure with clients receiving CBT and clients with anxiety (*p* = .41). Tukey’s post-hoc test revealed that participants had significantly stronger intentions to use behavioral activation for clients with depression (*M* = 4.97, *SD* = 1.48, *p* = .01) than clients with anxiety. No difference was observed between the strength of intentions to use behavioral activation among clients with depression and clients receiving CBT (*p =* .22) and clients with anxiety and clients receiving CBT (*p* = .73). Strength of intentions to use other CBT components did not differ among the clinical populations.

## Discussion

In this sample of community clinicians trained in CBT, we found that the strength of clinicians’ intentions to use different CBT components differed. This finding has immediate clinical implications. Given that intentions toward using some components were weak, while others were relatively strong, implementation strategies that target increasing high-fidelity use of CBT broadly may not be sufficient to increase clinician use of all CBT components. Consistent with hypotheses, compared with the strength of intentions to use structural components of CBT, intentions were relatively weak towards using intervention components, which may require more tailored implementation strategies. A related, methodological implication is that researchers querying clinicians about their thoughts regarding use of CBT should ask questions separately about different CBT components; traditionally, clinicians have been asked to report thoughts about CBT as a whole.

Intentions to use the structural components of CBT – agenda setting, planning and reviewing homework – were strongest and highly correlated with each other. Clinicians may view these as a suite of activities that they mostly intend or don’t intend to use. This was not the case for the CBT intervention components. Intentions to use cognitive restructuring and behavioral activation were highly correlated but intentions to use exposure was not correlated with either. Among the six components of CBT we asked clinicians about, intentions to use exposure were weakest and few clinicians endorsed that they “strongly agree” that they intend to use exposure. This is consistent with prior findings about clinicians’ attitudes toward and use of exposure, which are negative even when treating individuals with anxiety for whom exposure is most warranted [17].

These findings underscore variability in clinician CBT use in “real world” contexts and have important implications for implementation strategy selection [6]. Different implementation strategies may be needed when intentions are weak versus when they are strong. For example, implementation strategies should be developed to strengthen intentions to use exposure with clients experiencing anxiety. If clinicians are unlikely to use the CBT components for which intentions are strong, such as reviewing homework, implementation strategies should be designed to help clinicians act on their intentions. For example, if intentions are strong but clinicians forget to review homework, strategies to help clinicians remember may be most needed.

One possible explanation for these findings is that clinicians trained in CBT strongly intend to structure their sessions according to key CBT principles (e.g., setting agendas, assigning homework) because they judge these strategies as relatively easy to implement. In addition, or alternatively, they may feel they are expected to use these strategies, and/or that other clinicians frequently use these strategies; these perceived norms could strengthen their intentions. When it comes to intervention strategies that are more complex, or where perceived norms and attitudes may dictate strategies such as exposure are less acceptable or beneficial, clinicians may select strategies that best fit their practice style or that they feel most comfortable using, regardless of diagnosis or the evidence-base. Further studies that elucidate the extent to which intentions are influenced by perceived norms, attitudes and self-efficacy are needed to inform selection of implementation strategies. For example, in some instances you may need to select strategies, such as training and consultation, to increase self-efficacy. In cases where weak intentions are driven by perceived norms, establishing clinical champions may be helpful.

There were few differences in the strength of intentions across CBT, anxiety, and depression clinical groups. Consistent with hypotheses, no differences were found in the strength of intentions to use the structural components of CBT by clinical groups. Differences observed in intentions to use specific intervention components, such as exposure and behavioral activation, were appropriate for treatment groups, also as hypothesized. This suggests most clinicians differentiate exposure as being appropriate for anxiety and behavioral activation as being appropriate for depression. Taken together, these findings indicate that there may not be as much need for researchers to query separately about treatment groups when assessing clinicians’ thoughts regarding the use of CBT components.

## Conclusions

When studying determinants of CBT use and designing interventions to increase use, variability in intentions should be taken into account. Given the malleability of intentions, they should be targeted when developing implementation strategies to increase clinician EBP use. Additionally, researchers querying clinicians about their thoughts regarding use of CBT should ask questions separately about different CBT components rather than asking about CBT as a whole.

## Data Availability

The dataset supporting the conclusions of this article is available from the authors on reasonable request.

## Abbreviations

ANOVA: Analysis of variance
CBT: Cognitive-behavioral therapy
DBHIDS: Philadelphia Department of Behavioral Health and Intellectual disability Services
EBP: Evidence-based practice
ICC: Intraclass correlations
Penn BCI: University of Pennsylvania’s beck community initiative
REDcap: Research Electronic Data Capture
